# Brain activation during the expectations of sensory experience for cutaneous electrical stimulation

**DOI:** 10.1016/j.nicl.2018.06.022

**Published:** 2018-06-20

**Authors:** Won-Mo Jung, Yeonhee Ryu, Hi-Joon Park, Hyejung Lee, Younbyoung Chae

**Affiliations:** a Acupuncture & Meridian Science Research Center, College of Korean Medicine, Kyung Hee University, Seoul, Republic of Korea; b KM Fundamental Research Division, Korea Institute of Oriental Medicine, Daejeon, Republic of Korea

**Keywords:** Anterior insula, Expectation, fMRI, Pre-supplementary motor area, Somatosensation

## Abstract

The brain actively interprets sensory inputs by integrating top-down and bottom-up information. Humans can make inferences on somatosensation based on prior experiences and expectations even without the actual stimulation. We used functional magnetic resonance imaging to investigate the neural substrates of the expectations of the sensory experience of cutaneous electrical stimulation on acupoint without actual stimuli. This study included 22 participants who wore sticker-type electrodes attached on three different acupoints on different body regions: CV17 (chest), CV23 (chin), and left PC6 (arm). Participants evaluated *de qi* sensations after they expected electrical stimulation on those points in random order without actual stimulation. All stimuli were presented with corresponding visual information of the stimulation sites. The control condition included the same visual information but outside the body. The expectations of cutaneous electrical stimuli without actual stimulation on three acupoints resulted in greater *de qi* sensation compared to the control condition. Cognitive components of cutaneous electrical stimulation exhibited greater brain activation in the anterior insula, pre-supplementary motor area, and secondary somatosensory area. The expectations of acupuncture stimulation exhibited a distinct experience of somatosensation as well as brain activations in insula and pre-supplementary motor area. Our findings suggest that the sensory experience of the pseudo-cutaneous stimulation may be derived from the predictive role of the salience network in monitoring internal and external body states.

## Highlights

• The pseudo-cutaneous stimulation can elicit remarkable somatic sensations.
• Insula and pre-SMA are involved in the expectations of sensory experience.
• Somatic sensation from pseudo-stimulation is influenced by top-down information.

## 1 Introduction

In clinical practice, therapeutic cutaneous stimulation including acupuncture and transcutaneous electrical nerve stimulation (TENS) is a complex, ritualistic somatosensory intervention with multiple components including diagnosis and palpation followed by a resting stage after a needle insertion (Conn, Marshall, Yadav, Daly, and Jaffer, 1986; Hrobjartsson and Gotzsche, 2001; Kaptchuk, 2002; Langevin et al., 2006; Liu, 2009). Sham acupuncture, which does not entail skin penetration, aims to minimize the amount of physical stimulation in the course of the acupuncture ritual without altering other components (Chae, 2017; Chae et al., 2011a; b). However, several randomized controlled trials have reported that sham acupuncture is as effective as regular acupuncture, which has inspired placebo studies (Linde et al., 2005; Vase et al., 2013). Placebo needles can create a similar degree of somatosensation as verum acupuncture (Chae, 2017; Lee et al., 2014; Salih, Baumler, Simang, and Irnich, 2010). Even with mock laser acupuncture (Salih et al., 2010) or phantom acupuncture (Lee et al., 2014), where no physical stimulation is delivered, participants perceive a similar intensity of sensation to real laser acupuncture. Taken together, these results suggest that sensory experiences from acupuncture stimulation are not only the product of bottom-up modulation of simple needling but also the reciprocal interaction of top-down modulation of the brain (Chae et al., 2015). Therefore, we can expect that the cognitive component of cutaneous electrical stimulation alone induces a perceived sensation, even without an afferent somatosensory signal.

In acupuncture studies that have used brain imaging, activation of the salience network following acupuncture stimulation has been consistently reported (Bai et al., 2009; Chae et al., 2013; Chae et al., 2015; Fang et al., 2009; Napadow et al., 2009a; b; Napadow et al., 2013; Pariente, White, Frackowiak, and Lewith, 2005). Importantly, salience network activation is involved in the component of enhanced attention to a certain part of the body, even without actual stimulation (Chae et al., 2015). The brain actively interprets sensory inputs by integrating top-down and bottom-up information. In recent years, the predictive coding theory has suggested that the brain continually generates a model of the external environment and internal state, predicts incoming signals based on the model, and updates the model based on error between predicted and incoming signals (Friston, 2010; Knill and Pouget, 2004; O'Reilly, Jbabdi, and Behrens, 2012). The idea of predictive coding can facilitate understanding of the underlying neural processes of various cognitive functions from emotion to placebo analgesia (Buchel, Geuter, Sprenger, and Eippert, 2014; Schwarz, Pfister, and Buchel, 2016; Seth, 2013). According to predictive coding theory, perception is generated through the interplay between bottom-up sensory input and top-down prediction (O'Reilly et al., 2012; Schwarz et al., 2016). This can help explain illusory percepts, including phantom pain and tinnitus, and the salience network, which consists of the anterior insula and the anterior cingulate cortex, is a decisive contributor to these phenomena (De Ridder, Elgoyhen, Romo, and Langguth, 2011; De Ridder, Vanneste, and Freeman, 2014). Thus, it is expected that the sensory experience of placebo acupuncture, even in the absence of a physical stimulus, can be understood from this point of view.

We investigated the psychophysical and psychophysiological responses to the cognitive component of cutaneous electrical stimulation. We used functional magnetic resonance imaging (fMRI) to evaluate somatosensation and brain responses to the expectations of sensory experience regarding cutaneous electrical stimulation. Figurative visualization implied the delivery of stimulation without invasive cutaneous stimuli. We hypothesized that expectation of cutaneous electrical stimulation may induce prominent somatosensation in the absence of an afferent somatosensory signal, and that the salience network in the brain, which has a role in the prediction of error-based probabilistic learning of bodily sensation, is involved in the expectations of that sensory experience.

## 2 Methods

### 2.1 Participants

A total of 22 healthy human volunteers (23.7 ± 3.3 years old; 8 females) were recruited via advertisements among students of Kyung Hee University and Korea University. None of the participants had any history of neurological, psychiatric, or other major medical problems, and no participants used medications at the time of the study. Subjects with specialized knowledge of acupuncture were excluded from the participation. Participants were not allowed to drink alcohol or caffeine or to take any medications on the day of the experiment. All participants provided written informed consent before the experiments. The Institutional Review Board at Korea University approved all study procedures.

### 2.2 Experimental design and procedure

**Fig. 1:**
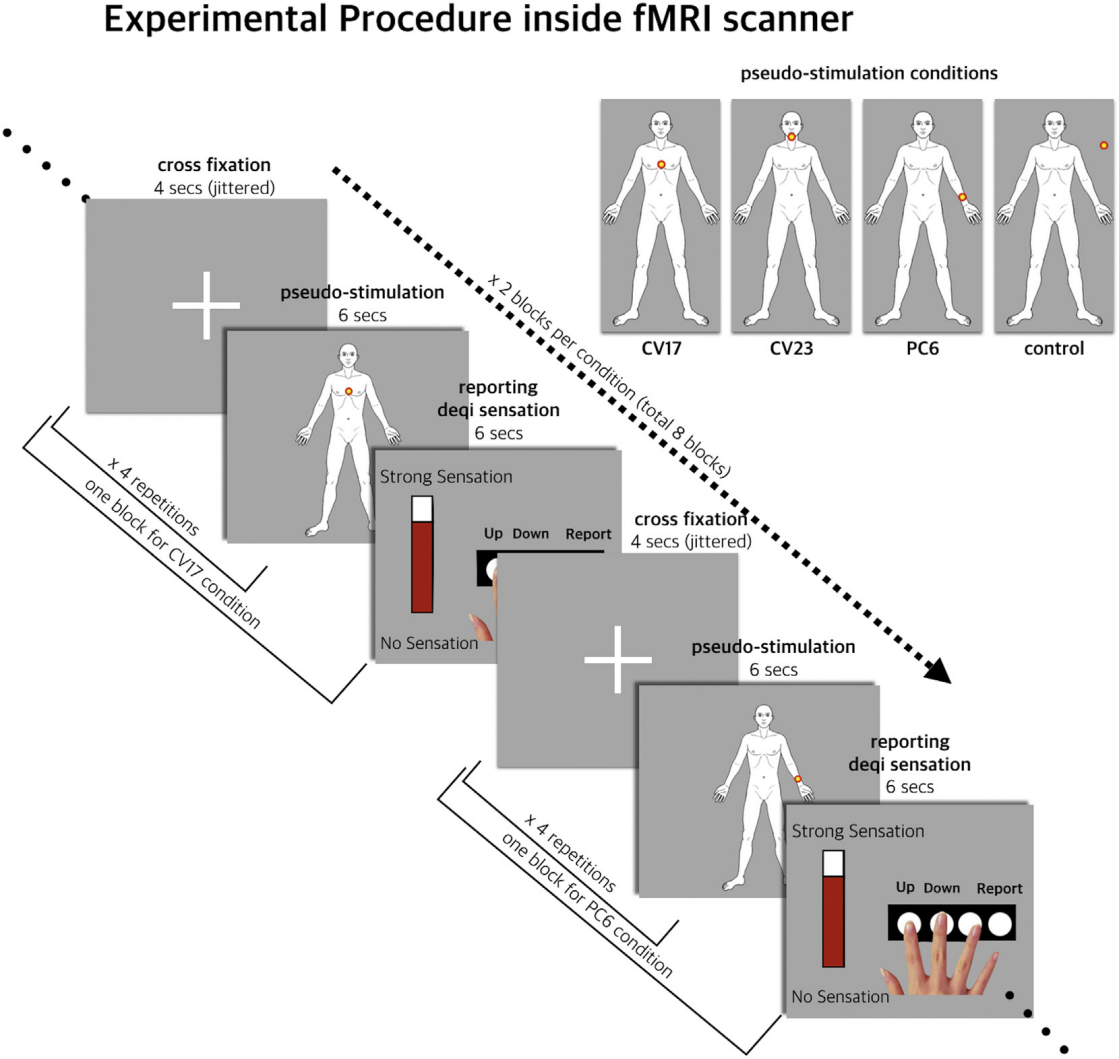
Experimental design and procedure. In one block, visual sign of pseudo-stimulation after cross fixation was presented four times, and the total intensity of somatosensation for four stimuli was evaluated. Pseudo-electrical stimulation consisted of three conditions, CV17 (chest), CV23 (chin), and PC6 (left forearm), which imply pseudo-stimulation by electric current on each acupoint. The presentation of a flickering dot on the human body template guided the timing of pseudo-stimulation to participants. The dot flickered outside the body in the control condition, which guided no stimulation to participants. After four consecutive pseudo-stimulations, a vertical bar was provided on the screen to collect an assessment of somatosensation during pseudo-stimulation. Participants were instructed to adjust the height of the vertical bar to indicate the intensity of the somatosensation. Scanning for brain structure proceeded after eight blocks.

To deliver the cognitive component of cutaneous electrical stimulation without afferent somatosensory input, pseudo-electrical stimulation was designed as an experimental stimulus. Before fMRI scanning, sticker-type electrodes designed with a carbon-filled plastic snap (ClearScan; Vermed Inc., NY, USA) were attached to three different acupoints: CV17 (chest), CV23 (chin), and left PC6 (forearm) (Fig. 1). All participants were informed that the nature of the study was to investigate the neural substrates of a newly developed tool called the Transdermal Micro-electric Current Stimulation Device (TMCSD), an advanced form of electro-acupuncture stimulation. Participants were also instructed that they would receive a very weak electric current through the attached electrodes and that the delivery of stimulation would be signaled using visual information. The sticker-type electrodes were not connected to any device inside the fMRI scanner. Participants did not receive any actual electric stimulation.

According to the position of the flickering dot, pseudo-stimulation was classified into three conditions: CV17, CV23, and PC6, as well as the control. To signal the delivery of pseudo-stimulation, a dot with gradated color from yellow to red flickered six times with a cycle of 1 s on the front of the human body. The location of the flickering dot varied among four positions: CV17 (chest), CV23 (chin), PC6 (forearm), and outside the body. The location on the human figure indicated the location of the acupoint to receive pseudo-stimulation. When the dot flickered outside the body, it indicated no electric current was transmitted (control condition). This information was shown in the fMRI scanner using MRI compatible visual goggles (800 × 600 pixels; refresh rate: 85 Hz; signal transfer: fiber-optic cable through waveguide; NordicNeuroLab, Bergen, Norway). Inside the fMRI scanner, participants underwent eight blocks in total, two for each condition. In one block, pseudo-electrical stimulation for 6 s after cross fixation was presented four times, and then the intensity of *de qi* sensation for four consecutive stimuli was evaluated. Durations of the presentations of the cross fixation were jittered using a uniform distribution ranging from 2800 ms to 5200 ms.

To collect an assessment of *de qi* sensation during pseudo-stimulation, a vertical bar was provided on the screen. Participants were instructed to adjust the height of the vertical bar to indicate the intensity of the *de qi* sensation using the MRI compatible button box (Current Design™, Philadelphia, USA). On the left side of the vertical bar, information about the height of the bar was provided as a number from 0 to 100. To avoid the formation of instructional bias, we did not provide participants with an explanation for what the bodily sensation from stimulation will be. Instead participants were instructed to use VAS to express the intensity of the sensation, which covers all sorts of possible bodily senses together. The participants were told that the number 100 indicates the greatest intensity of *de qi* sensation imaginable and that 0 indicates no sensation. *De qi* sensation in this study included a range of bodily sensations from acupuncture stimulation, including dull, numb, and heavy (Jung et al., 2016). Each rating screen appeared for 6 s and the participants were asked to make their decision before the end of this period. When the vertical bar was presented, the initial height of the bar was randomly chosen from 0 to 100 using uniform distribution. Scanning for brain structure proceeded after the eight blocks.

After scanning, participants were asked to mark the locations of their bodily sensations for each condition of pseudo-electrical stimulation on a given somatotopic map using a bodily sensation map application, which presents a template of the human body as two-dimensional frontal images (1536 × 2048 pixels; http://cmslab.khu.ac.kr/downloads/bsm) with an iPad (Apple Inc.; Cupertino, CA, USA) (Jung, Lee, Lee, and Chae, 2017a; b; Jung et al., 2016). The spatial patterns of pseudo-stimulation-induced sensations were analyzed by the pixel-wise evaluation of associations between each condition and the spatial configuration of the bodily sensation map (Jung et al., 2016). The general pattern of sensation was visualized with a 10% response rate threshold.

### 2.3 fMRI acquisition

Structural and functional imaging was completed on a Magnetom Trio 3 T magnetic resonance scanner (Siemens, Erlangen, Germany). As an anatomical reference, a three-dimensional T1-weighted magnetization-prepared rapid gradient echo (MPRAGE) image dataset was obtained (TR = 2000 ms, TE = 2.37 ms, flip angle = 9°, field of view = 240 × 240 mm^2^, voxel size = 0.9 × 0.9 × 1.0 mm^3^, and 192 slices). Blood oxygen level-dependent fMRI of the whole brain was conducted using an echo planar imaging (EPI) sequence (TR = 2000 ms, TE = 30 ms, flip angle = 90°, field of view = 240 × 240 mm^2^, voxel size = 3.8 × 3.8 × 4.0 mm^3^, and 37 slices). Stimulus presentation and response logging were completed using the PsychToolbox program in Matlab (MathWorks, Natick, MA, USA).

### 2.4 fMRI analysis

As this study investigated the neural substrates of the sensory experience from pseudo-stimulations, we excluded two participants who did not express any *de qi* sensations in the fMRI analysis. Preprocessing was performed with the Analysis of Functional NeuroImages (AFNI) software package (Cox, 1996). EPI time series data were corrected for slice timing slice timing, and then concatenated and aligned to T1 anatomy image, transformed to a common space, Talairach space (Paus et al., 1996), corrected for motion by registering to the volume with the minimum outlier fraction, spatially blurred using a 6 mm full-width-at-half-maximum (FWHM) Gaussian filter, resampled to 3 mm isotropic resolution, and scaled to have mean of 100 for each voxel. Head movement during the scanning session was assessed prior to the movement correction to the fMRI data.

fMRI data were analyzed at the individual subject level using the timing from all stimuli in a multiple linear regression using a gamma variate hemodynamic response function. Regressors to account for brain activity not related to the pseudo-electrical stimulation included the timing of evaluation of intensity and head motion parameters. The six motion-correction parameters of head movement assessed from the realignment procedure were entered as covariates of no interest. The regressors of interest were used to model the unconditioned fMRI signal response to three conditions of pseudo-stimulation, including CV17, CV23, and PC6, and the control condition. This resulted in 11 regressors that were fitted to scan time courses using the AFNI program 3dDeconvolve (Cox, 1996).

To evaluate a common neural response to pseudo-stimulation, a contrast image was generated between an average coefficient of the three pseudo-stimulation conditions and the coefficient of the control condition. Cluster threshold criteria were determined by Monte Carlo simulations, which resulted in a family wise error (FWE)-corrected significance threshold of *p* < .05 (de Andrade et al., 2006; Forman et al., 1995) using the 3dClustSim program at voxel-wise *p*-value <.001. The spatial smoothness of the data was evaluated by a modern approach that estimates a non-Gaussian spatial autocorrelation function leading to greatly reduced false-positive rates. The actual smoothness of the data was determined using a modified version of AFNI's 3dFWHMx with the auto-correlation function (Cox et al., 2017a, Cox et al., 2017b).

## 3 Results

### 3.1 Psychophysical responses to the cognitive component of cutaneous electrical stimulation

**Fig. 2:**
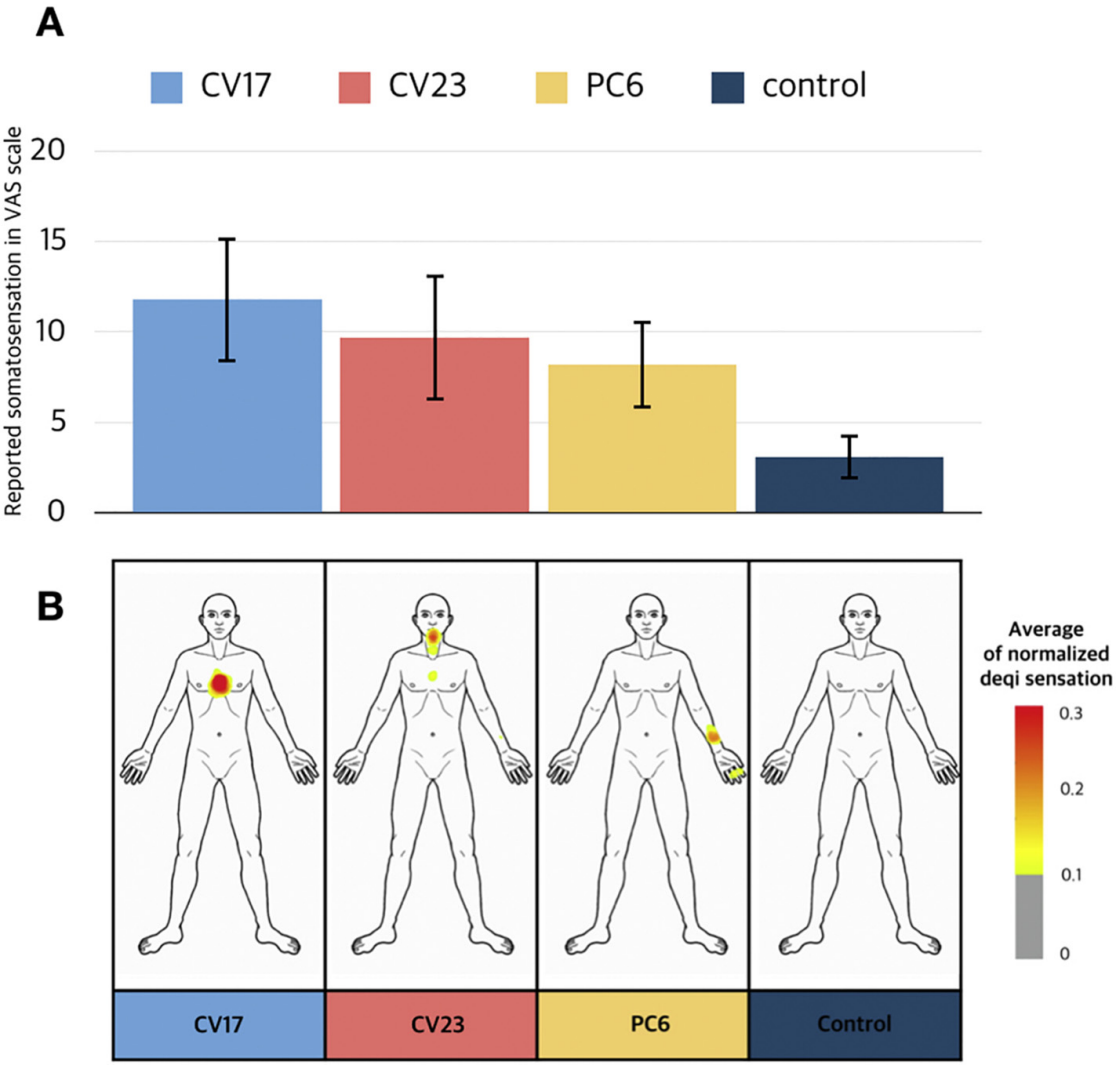
Psychophysical responses to the cognitive component of cutaneous electrical stimulation. The average somatosensation of each condition is exhibited in a bar graph (A). All pseudo-stimulation conditions elicited significantly greater *de qi* sensation than the control condition. All pseudo-electrical stimulations induced significantly greater intensity of *de qi* sensation compared to the control condition (CV17 vs. control: *t* = 3.06, *p* = .005; CV23 vs. control: *t* = 2.159, *p* = .042, PC6 vs. control: *t* = 2.341, *p* = .029). The somatotopic patterns of bodily sensation in each condition on a front-postured human body template are shown (B). The body regions showing somatosensation for each pseudo-stimulation condition matched well with the stimulated acupoint locations, while no sensation was shown in the control condition. The general distributed pattern of sensation was visualized with a 10% response rate threshold.

The *de qi* sensation was 11.8 ± 3.5 (mean ± standard error of the mean) for CV17, 9.7 ± 3.5 for CV23, 8.2 ± 2.4 for PC6, and 3.1 ± 1.2 for the control condition. A paired *t*-test revealed that all pseudo-stimulations induced significantly greater intensity of *de qi* sensation compared to the control (CV17 vs. control: *t* = 3.06, *p* = .005; CV23 vs. control: *t* = 2.159, *p* = .042, PC6 vs. control: *t* = 2.341, *p* = .029) (Fig. 2A).

The spatial patterns of stimulation-induced sensation reported after the experimental procedures were visualized on the body template. The body regions showing *de qi* sensation for each cognitive acupuncture condition matched well with the stimulated acupoint locations, while no sensation was shown on any part of the body regions in the control condition (Fig. 2B).

### 3.2 Psychophysiological responses to the cognitive component of cutaneous electrical stimulation

**Fig. 3:**
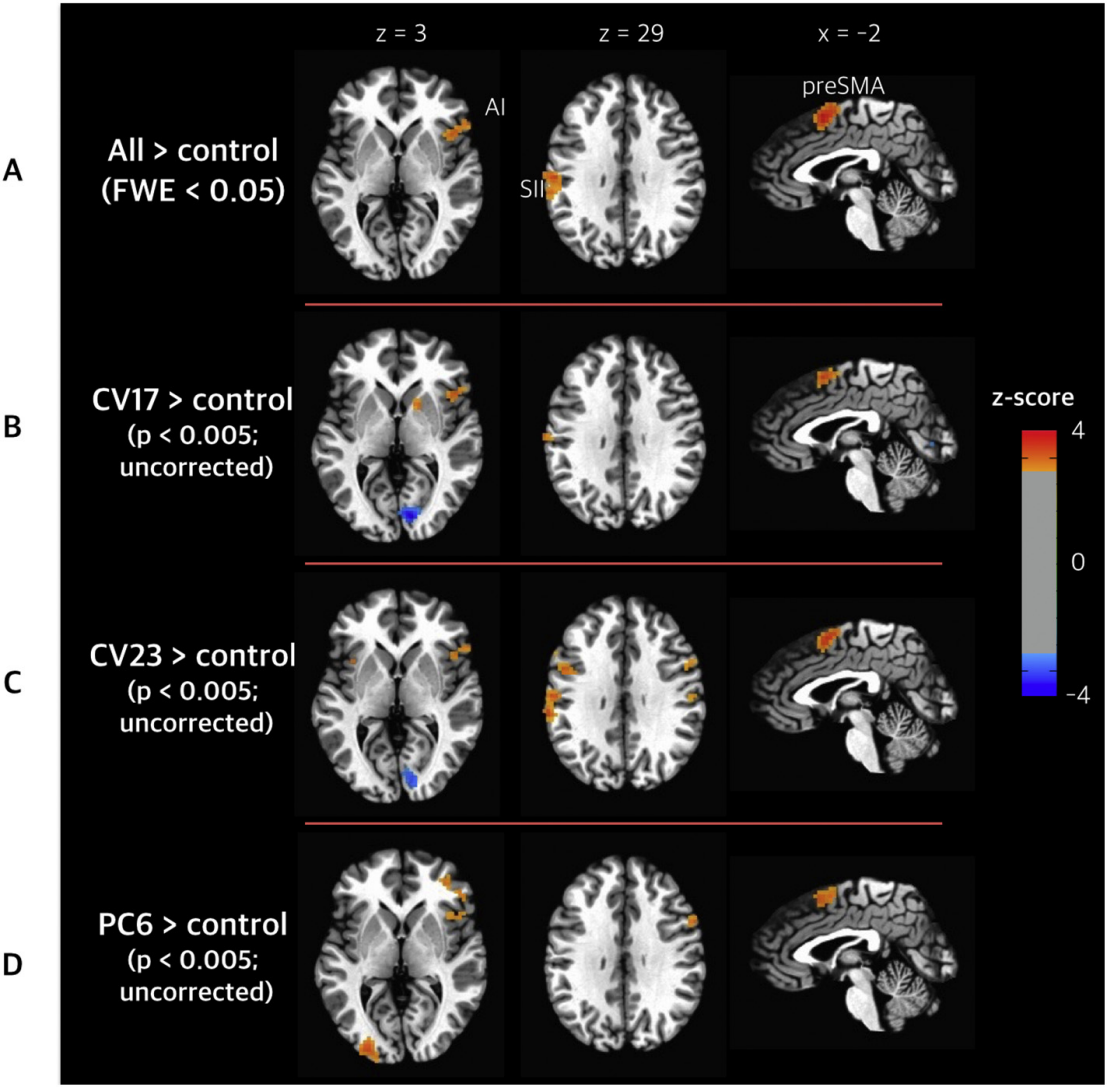
Psychophysiological responses to the cognitive component of cutaneous electrical stimulation. All three pseudo-stimulations commonly exhibited brain activation in the right anterior insula, right inferior frontal gyrus (rIFG), pre-supplementary motor area (pre-SMA), and secondary somatosensory cortex (SII) (*p* < .05, FWE-corrected). The common functional magnetic resonance imaging activation map (A) for pseudo-stimulation and the activation pattern of each condition (B, C, and D) in the same brain section. Statistical t-maps of all acupoint conditions over the control condition are overlaid on an average structural image, and the significance threshold was set to *p* < .005 (uncorrected) for visualization purposes only.

To identify common brain responses to all pseudo-stimulation compared to the control condition, we performed a group-level analysis on the differences between the average coefficients of the three cognitive acupuncture conditions and the coefficient of the control condition. All three pseudo-stimulations exhibited significantly greater brain activation compared to control condition (*p* < .05; FWE-corrected) in the right anterior insula, right inferior frontal gyrus (rIFG), pre-supplementary motor area (pre-SMA), and secondary somatosensory cortex (SII), which suggests that the part of the salience network in the brain is involved in the expectation of the sensory experience of cutaneous electrical stimulation, even without actual stimuli (Fig. 3). Table 1 provides a summary of the fMRI-activated clusters for cognitive acupuncture.

**Table 1:** Common brain responses to the expectations of sensory experience for pseudo-electrical stimulation.

Activation	Location		Peak *Z*-score	Voxels	Coordinates of peak voxel in Talairach space (right anterior inferior; RAI coordinate)		
					X	Y	Z
Increased activation	Pre-supplementary motor area	left	3.9	148	−1.5	−7.5	53.5
	Right anterior insula, right inferior frontal gyrus	right	3.7	127	55.5	19.5	26.5
	Secondary somatosensory cortex	left	4.1	114	−40.5	−19.5	14.5

All peaks have a corrected *P* value <.05 (FWE-corrected).

## 4 Discussion

We showed that pseudo-electrical stimulation, figurative visualization implying the delivery of stimulation without actual stimulation, elicited significant but weak *de qi* sensation in the body area around the intended acupoint. The cognitive component of cutaneous electrical stimulation was associated with greater brain activation in the anterior insula, pre-SMA, and SII. Our findings imply that the sensory experience of the cognitive component of cutaneous electrical stimulation may be derived from the predictive role of the salience network in monitoring internal and external body states.

The cognitively induced somatosensation observed in our study corroborates studies that have reported remarkably similar *de qi* sensation between verum and sham acupuncture with regard to intensity, quality, and somatotopic pattern (Beissner et al., 2015; Lee et al., 2014; Salih et al., 2010). *De qi* sensation, the somatosensation elicited by acupuncture stimulation, has been asserted to be a useful predictor of successful acupuncture treatment (Spaeth et al., 2013; Zhao et al., 2017). Furthermore, cognitive factors in acupuncture stimulation, such as a patient's perception and expectations, can influence acupuncture analgesia (Vase et al., 2013). These cognitive components of acupuncture stimulation are distinguished from pharmacological and other physical placebos (Kaptchuk, Goldman, Stone, and Stason, 2000), as well as in the effective aspect (Linde, Niemann, and Meissner, 2010); one distinction would be the intimate association between the cognitive processes of somatosensation, such as body awareness (Chae et al., 2014; Chae et al., 2015) and bodily attention (Chae et al., 2015). Considering the importance of the perceived sensation of acupuncture stimulation, we can expect that the expectation of the sensory experience plays a pivotal role in *de qi* sensation from therapeutic cutaneous stimulation including acupuncture.

In the present study, the expectation of cutaneous electrical stimulation exhibited greater activation in the anterior insula, pre-SMA, and SII in the brain. The salience network is known as an intrinsically connected brain network consisting of bilateral anterior insula (AI) and dorsal anterior cingulate cortex (dACC). However, the definition of the brain regions that constitute the Salience network differs slightly depending on the studies. Bonnelle's study (Bonnelle et al., 2012), which is one of the core studies of the salience network that found a reciprocal activation pattern between the salience network and the default network, reveals that the salience network consists of the medial wall of the frontal lobe area, including the ACC and preSMA, and the anterior insula. Thus, our findings are in line with previous brain imaging studies in which the salience network was activated by acupuncture stimulation (Bai et al., 2009; Chae et al., 2013; Chae et al., 2015; Fang et al., 2009; Napadow et al., 2009a; b; Napadow et al., 2013; Pariente et al., 2005). The AIC is involved in alertness (Sterzer and Kleinschmidt, 2010) and conscious awareness of the perception of pain (Albanese, Duerden, Rainville, and Duncan, 2007; Craig, 2002; Isnard, Magnin, Jung, Mauguiere, and Garcia-Larrea, 2011). Functional activity of salience network is known to be associated with the saliency or orienting effect evoked by noxious stimuli. By contrast, salience network is also commonly activated under non-painful conditions, such as tactile stimulation (e.g., sham acupuncture) (Napadow et al., 2009b; Napadow et al., 2013) or focusing bodily attention (Chae et al., 2015). In a recent study, Ostwald et al. applied the Bayesian stimulus probability learning algorithm to the entire EEG period and found that the inferior frontal gyrus adjacent to the AIC, medial cingulate cortex (MC), and SII encodes the delivery of odd stimuli during the human somatosensory processing of the violation of prior expectations by means of somatosensory mismatch (Ostwald et al., 2012). In addition, a recent computational modeling study described the Bayesian updating of knowledge driven by the predictive coding theory as the most plausible model to the human somatosensory system (Lieder, Daunizeau, Garrido, Friston, and Stephan, 2013). The theory of predictive coding provides a suitable explanation for the generation of phantom percepts, such as illusory pain and tinnitus, to reduce uncertainty and make sense of the world (De Ridder et al., 2014). Taken together, these findings and our results suggest that brain activation in the AIC and MC in response to pseudo-cutaneous stimulation may be associated with expectations of the sensory experience.

In the early days of brain science, the visceral response, which appears when electrically stimulating the insula, led people to perceive the insula as a visceral brain (Penfield and Faulk Jr., 1955). Subsequent studies, however, reported that substantial somatosensory responses are also accompanied by intracranial electrical stimulation of the human insula (Pugnaghi et al., 2011; Stephani, Fernandez-Baca Vaca, Maciunas, Koubeissi, and Luders, 2011). A tract tracing study in monkeys revealed that the mid-posterior granular insula is one of the main cortical areas connected to spinothalamic tracts (Dum, Levinthal, and Strick, 2009) that transmits pain, temperature (Craig, 2002), and affective touch (Mcglone, Wessberg, and Olausson, 2014). The AIC is interconnected with the posterior insula and somatosensory cortex with regard to both anatomical (Cerliani et al., 2012) and functional (Chang, Yarkoni, Khaw, and Sanfey, 2013) connectivity. Based on the transmitted afferent inputs, the AIC can play a pivotal role in the integration of interoceptive and exteroceptive signals, as well as in generating subjective feeling states (Craig, 2009; Critchley, Mathias, and Dolan, 2002; Harrison, Gray, Gianaros, and Critchley, 2010; Nagai, Critchley, Featherstone, Trimble, and Dolan, 2004). The AIC is also involved in processing information about uncertainty and awareness of error during decision-making (Eichele et al., 2008; Elliott, Friston, and Dolan, 2000; Grinband, Hirsch, and Ferrera, 2006; Klein et al., 2007; Paulus, Rogalsky, Simmons, Feinstein, and Stein, 2003). Regarding predictive coding theory, brains engage in a probabilistic learning of the external and internal environment according to prediction errors (Friston and Kiebel, 2009; Lieder et al., 2013; Seth and Friston, 2016). Thus, the comparator calculating prediction error is pivotal in the prediction error-based learning system. Various models have recently suggested that the AIC connects the feeling of agency by integrating interoceptive and exteroceptive signals by a parallel predictive-coding mechanism (Craig, 2009; Seth, Suzuki, and Critchley, 2011; Singer, Critchley, and Preuschoff, 2009). Considering the neurological function of the AIC, we assume that the activation of the AIC observed in our study represents the prediction error of expecting somatosensation from electric stimulation in the absence of an afferent signal.

In our study, pre-SMA was activated following pseudo-stimulation. In neuroimaging studies, the term posterior medial prefrontal cortex (pMFC) generally refers to the frequently coactivated brain regions, pre-SMA and aMCC, as well as the adjacent SMA, posterior dmPFC, and pMCC (Ullsperger, Danielmeier, and Jocham, 2014). This region corresponds to the agranular cortex and its cortical columns are characterized by less laminar differentiation (Barbas and Rempel-Clower, 1997; Shipp, Adams, and Friston, 2013; van den Heuvel and Sporns, 2013). It was recently suggested that the agranular cortices in the pMFC are involved in the prediction signal of bodily state based on neuroanatomical characteristics (Barrett and Simmons, 2015; Shipp et al., 2013). Although the ACC may make a significant contribution to the prediction of bodily state by constituting a salience network with the AIC, the specific function of the ACC appears to be distinct from that of the AIC. The AIC is related to awareness (Klein et al., 2007), while the ACC is more involved in the modulation of bodily state to meet behavioral demand based on action-outcome pairing (Alexander and Brown, 2011; Critchley, 2009; Critchley et al., 2003; Eichele et al., 2008; Klein et al., 2007; Pool and Ransohoff, 1949). In a previous study that explored Bayesian surprise of the human somatosensation system, prediction error-based learning of somatosensation was attributable to the medial cingulate cortex at a later processing step (around 360 ms) compared to the SII (140 ms) and AIC (250 ms) (Ostwald et al., 2012). We strongly believe that the activation of pre-SMA observed in our study may be related with the autonomic modulation based on the mismatch of somatosensation.

Several limitations to our study should be discussed. First, our experiments mainly focused on the examination of the neural substrates of pseudo-cutaneous stimulation on healthy volunteers. Further study is needed to explore the therapeutic actions of cognitively induced somatosensation in clinical situations, such as the modulation of pain or the autonomic nervous system, in addition to somatosensation. Second, electro-dermal activity is widely used as a biological marker of autonomic function, reflecting Bayesian surprise, in prediction error-based learning systems (Bach, Daunizeau, Kuelzow, Friston, and Dolan, 2011; Nagai et al., 2004; Studer, Scheibehenne, and Clark, 2016). In this study, however, autonomic responses such as skin conductance level could not be measured simultaneously with fMRI due to methodological limitations. Analyzing fMRI signals with skin conductance level would benefit investigations on the neural basis of cognitively induced sensory experiences. Third, we placed the stimulating dot outside of the body in our control condition. Someone may argue that placing the stimulation dot on the human body itself cause attention to the body part. Fourth, as we did not include any clinical outcome in this study, we cannot claim that sensory experience for cutaneous electrical stimulation can be compatible for the genuine electrical stimulations. Further studies are necessary to investigate the role of sensory experience in therapeutic effect. Finally, the sensory experiences from pseudo-electrical stimulation were relatively low in the current study. The intensity and spatial patterns of *de qi* sensation, however, were significantly different from the control condition in experimental settings where actual stimulation was absent and did not require the expectation of intense sensation through the instruction. As the prediction for somatosensation was constantly modified by Bayesian updating, further study is needed using computational modeling.

In conclusion, the cognitive component of cutaneous electrical stimulation elicited significant somatosensation in the body area around the intended acupoint in the absence of actual stimulation. Furthermore, the insula and the pre-SMA in the brain are involved in the sensory experience from pseudo-cutaneous stimulation. Given the important role of the salience network in the brain, our findings suggest that prediction error-based probabilistic learning of the salience network is involved in the sensory experience elicited by the cognitive component of cutaneous electrical stimulation.
